# Genome-wide analysis of respiratory burst oxidase homolog gene family in pea (*Pisum sativum* L.)

**DOI:** 10.3389/fpls.2023.1321952

**Published:** 2023-12-12

**Authors:** Minmin Liu, Yu Zhang, Ting Pan, Yuanyuan Li, Youheng Hong, Wenjie Chen, Yao Yang, Gangjun Zhao, Sergey Shabala, Min Yu

**Affiliations:** ^1^International Research Centre for Environmental Membrane Biology and Department of Horticulture, Foshan University, Foshan, China; ^2^Guangdong Key Laboratory for New Technology Research of Vegetables, Vegetable Research Institute, Guangdong Academy of Agricultural Sciences, Guangzhou, China; ^3^School of Biological Science, University of Western Australia, Crawley, WA, Australia

**Keywords:** reactive oxygen species, abiotic stress, plasma membrane, drought, salinity, cadmium, boron

## Abstract

Plant respiratory burst oxidase homologs (RBOHs) are key enzymes regulating superoxide production, which is important for plant development and responses to biotic and abiotic stresses. This study aimed to characterize the *RBOH* gene family in pea (*Pisum sativum* L.). Seven *PsRBOH* genes were identified in the pea genome and were phylogenetically clustered into five groups. Collinearity analyses of the *RBOHs* identified four pairs of orthologs between pea and soybean. The gene structure analysis showed that the number of exons ranged from 6 to 16. Amino acid sequence alignment, conserved domain, and conserved motif analyses showed that all seven PsRBOHs had typical features of plant RBOHs. The expression patterns of *PsRBOH* genes in different tissues provided suggested their roles in plant growth and organ development. In addition, the expression levels of *PsRBOH* genes under different abiotic stresses were analyzed *via* reverse transcription-quantitative polymerase chain reaction (RT-qPCR). The results demonstrated that *PsRBOH* genes exhibited unique stress-response characteristics, which allowed for functional diversity in response to different abiotic stresses. Furthermore, four PsRBOHs had a high probability of localization in the plasma membrane, and PsRBOH6 was localized to the plasma membrane and endoplasmic reticulum. The results of this study provide valuable information for further functional analysis of pea *RBOH* genes and their role in plant adaptation to climate-driven environmental constraints.

## Introduction

Abiotic stress tolerance of major staple crops was significantly weakened or even lost during their domestication (Palmgren et al., 2015; Rawat et al., 2022). Major abiotic stresses such as drought, salinity, flooding, and extreme temperatures are increasing due to the current climate changes driven by global warming (Liu et al., 2020). Therefore, improving abiotic stress tolerance in crops is critical for future food security.

Despite different plants having different mechanisms for adapting to abiotic stresses, these stresses increase superoxide levels in all plants, and the superoxide is transformed into reactive oxygen species (ROS) *via* different pathways in different cellular compartments (Sandalio et al., 2021). ROS were initially considered toxic byproducts of aerobic metabolism and deemed harmful to plants; however, their central role in complex adaptive signaling networks has been recently reported (Mittler et al., 2011). While excessive ROS accumulation is toxic, causing DNA backbone damage, protein and lipid oxidation, and inducing apoptosis (Patel et al., 2018; Wang et al., 2020), transient stress-induced ROS spikes are considered central to systemic signaling and adaptation in plants (Bostock et al., 2014; Zhang et al., 2022).

Superoxide can be produced in various cellular compartments, including cytosolic and apoplastic compartments. Respiratory Burst Oxidase Homologues (RBOH) play a key role in apoplastic superoxide production (Suzuki et al., 2011; Zhang et al., 2022). The first identified plant *RBOH* gene was *OsRBOHA* in rice (Groom et al., 1996). With the recent increase in whole-genome sequencing of more plant species, *RBOH* genes have been functionally characterized in various plant species. These plants include *Arabidopsis thaliana* (Torres et al., 1998; Torres et al., 2002), *Lycopersicon esculentum* (Sagi et al., 2004), *Olea europaea* (Jimenez-Quesada et al., 2019), *Glycine max* (Liu et al., 2019), *Brassica rapa* (Li et al., 2019), *Capsicum annuum* (Zhang et al., 2021), *Pyropia yezoensis* (Gui et al., 2022), and *Solanum melongena* (Du et al., 2023).

Previous studies showed that many plants have multiple RBOH members, and different members exhibit different expression profiles with distinct functions. For example, there are ten RBOHs in *Arabidopsis* (AtRBOHA-J) (Sagi and Fluhr, 2006) and nine in rice (OsNox1-9) (Wang et al., 2013). Additional studies showed that *AtRBOHD* and *AtRBOHF* were expressed throughout the plant, while *AtRBOHA-C*, *AtRBOHG* and *AtRBOHI* were specifically expressed in the roots. Moreover, *AtrbohH* and *AtrbohJ* were specifically expressed in pollens and are thus required for normal pollen tube growth (Sagi and Fluhr, 2006; Kaya et al., 2014). *OsNox1-2*, *OsNox5-6*, and *OsNox9* were ubiquitously expressed, while *OsNox3*, *OsNox4*, *OsNox7*, and *OsNox8* showed tissue-specific expression in rice (Wang et al., 2013). Moreover, *OsNox9* were associated with root development (Takeda et al., 2008; Yamauchi et al., 2017).

These studies also provided evidence for the involvement of RBOHs in plant signal transduction during abiotic stress responses and development. Plant RBOHs are involved in several signaling pathways, including Ca^2+^-dependent protein kinases (CDPKs) (Dubiella et al., 2013), mitogen-activated protein kinases (MAPKs) (Asai et al., 2008), receptor-activated C-kinases (RACKs) (Nakashima et al., 2008), phosphatidylinositol, phospholipase Dα1and phosphatidic acid (Zhang et al., 2009), nitric oxide (NO) (Delledonne et al., 2002), cGMP (Li et al., 2011), extracellular ATP-mediated signaling pathways (Song et al., 2006), and hormonal signaling networks (abscisic acid, salicylic acid, jasmonic acid, and ethylene) (Overmyer et al., 2003). AtRBOHD and AtRBOHF regulate ABA-mediated stomatal closure (Kwak et al., 2003) and salt stress tolerance in *Arabidopsis* (Chung et al., 2008; Xie et al., 2011).

As a classic model plant and the second most important grain legume, pea (*Pisum sativum* L., 2n = 14) has been widely used in genetics and developmental biology studies since Mendel (Kreplak et al., 2019; Chen et al., 2022; Chen et al., 2023; Li et al., 2023). Pea has become an important legume crop and green vegetable favored by many people worldwide. Fresh pea is rich in soluble protein, starch, carotenoid, and flavonoids (Yang et al., 2022). However, pea is sensitive to most common abiotic stresses (e.g., drought, cold, high temperatures, and salinity), which cause massive yield losses.

Given the critical role of RBOH in plant adaptive responses to abiotic stresses, in this work we aimed to provide a comprehensive analysis of pea *RBOH* genes. This study analyzed the chromosomal distribution, developmental evolution, gene structure, conserved domains, subcellular location, and description of cis-acting elements of pea *RBOH* genes. We also studied the tissue specificity and expression patterns of pea *RBOH* genes under different abiotic stresses. The reported data provide valuable information for an in-depth understanding of the biological functions of the *RBOH* gene family in pea, paving the way for improving its abiotic stress tolerance.

## Materials and methods

### Identification of the *PsRBOH* genes in pea

The DNA sequences and annotation files of the pea genome were downloaded from the Pea Genome Database (https://www.peagdb.com/). In addition, 17 soybean, 8 tomato, and 10 *Arabidopsis* RBOH protein sequences were downloaded from the phytozome (https://phytozome-next.jgi.doe.gov/), Sol Genomics Network (https://www.sgn.cornell.edu/) and TAIR (www.arabidopsis.org/) databases, respectively. *Arabidopsis* is a model plant widely used in various plant studies, including gene family identification studies. Tomato is a horticultural crop that has been widely studied and is one of the most widely eaten vegetables in the world. Its molecular biology research is relatively in-depth. Soybeans, like peas, are important leguminous plants with high protein levels. The domain sequences (HMM model file) of the NADPH oxidase (PF08414), NAD binding (PF08030), FAD binding (PF08022), and Ferric reductase domains (PF01794) were downloaded from the Pfam database (http://pfam.xfam.org/) and used as the seed files to search the RBOH proteins in the pea genome file *via* a hidden Markov model (HMM) search (e-value 0.01). The conserved domains were verified using CD-Search (https://www.ncbi.nlm.nih.gov/Structure/cdd/wrpsb.cgi).

### Analysis of the chromosomal location, phylogenetics, collinearity, gene structure, conserved motifs, and cis-elements

The online software MG2C (http://mg2c.iask.in/mg2c_v2.0/) was used for the chromosome localization analysis. The phylogenetic tree was constructed using the IQ-Tree Wrapper program in TBtools software (Chen et al., 2020) with 1000 bootstrap replicates. The collinearity between pea and soybean sequences was determined using TBtools software (Chen et al., 2020). The Gene Structure Display Server (http://gsds.gao-lab.org) was used to generate the gene structure map, and the online software MEME (http://meme-suite.org/tools/meme) was used to search for conserved motifs with the maximum conserved motif search value set to 10. The 2000-bp sequences upstream of *PsRBOH* genes were obtained from the pea genome database and used as the promoter, and the cis-regulatory elements of these promoters were identified using PlantCARE (http://bioinformatics.psb.ugent.be/webtools/plantcare/html/).

### Plant materials, stress treatments and tissue expression analysis

*P. sativum* cultivar Zhongwan6 (ZW6) were obtained from the pea seed breeding center in China (Gu’an, Hebei). The seeds were germinated in a liquid culture using a quarter (1/4)-strength modified Hoagland nutrient solution (pH 5.5) in a growth chamber under 25 ± 2°C with a 16 h/8 h light/dark cycle. The four-leaf stage seedlings were treated with 1/4-strength modified Hoagland nutrient solution [Ca (NO_3_)_2_·4H_2_O 236.25 mg/L+KNO_3_ 126.25 mg/L+ NH_4_ NO_3_ 20 mg/L+ KH_2_PO_4_·2H_2_O 34 mg/L+ MgSO_4_·7H_2_O 123.25 mg/L, pH 5.5] supplemented with 100 mM NaCl for the salt-stress treatment, 10% (w/v) polyethylene glycol (PEG) for the drought stress treatment, and 6 μM CdCl_2_ for the cadmium (Cd) treatment. For the heat and cold stress, the seedlings were treated with 1/4-strength modified Hoagland nutrient solution (pH 5.5) at 38°C and 4°C, respectively. For the low-boron stress treatment, seedlings were initially pretreated with the modified Hoagland nutrient solution containing 0.025 μM boric acid (H_3_BO_3_) (B treatment) at the first stage of culture to diminish variations in B concentrations in seeds. Subsequently, the low-boron-treated plants were treated with the modified Hoagland nutrient solution containing 0.025 μM H_3_BO_3_, and the controls were treated with the modified Hoagland nutrient solution with 25 μM H_3_BO_3_ at the four-leaf-stage seedlings (Li et al., 2018). The treatments were conducted in triplicates with five seedlings per replicate. The leaves of treated seedlings were sampled after 0.5, 3, 6, 12, 24, 36, 48, and 72 h of stress treatment. Pea roots, leaves, stems, flowers, tendrils, one-week-old pea seeds, one-week-old pod, and two-week-old pea seeds were sampled for tissue expression analysis. All the harvested samples were frozen in liquid nitrogen and stored at −80°C until analysis. Each treatment had three independent biological replicates.

### RNA isolation and RT-qPCR analysis

Total RNA was isolated from the samples using FreeZol Reagent R711 (Nanjing Vazyme Biotech Co., Ltd.), according to the manufacturer’s instructions. Briefly, 50 mg of the ground sample was added into 500 μl of FreeZol Reagent for lysis. The lysate was centrifuged, and the supernatant was collected. A dilution buffer was added to the supernatant, and the mixture was precipitated with isopropanol. After centrifugation, the supernatant was discarded, and the pellet was washed with 75% ethanol and dissolved in RNase-free double distilled water (ddH_2_O). The quality and concentrations of the isolated RNA samples were determined *via* 1% agarose gel electrophoresis and a NanoDrop 2000 Spectrophotometer (Thermo Fisher Scientific, Wilmington, DE, USA). Reverse transcription PCR was conducted on a QuantStudio™ 6Flex Real-Time PCR System (Applied Biosystems™, Carlsbad, CA, USA) using HiScript^®^ III RT SuperMix for qPCR with (+gDNA wiper) (R323-01) (Vazyme, Nanjin) and ChamQTM Universal SYBR^®^ qPCR Master Mix (Q711) (Vazyme). Three technical replicates were set for each biological sample, and the reaction conditions were as follows: 95°C for 30 s, followed by 40 cycles at 95°C for 10 s and 65°C for 20 s. A melting curve was generated by cooling from 95°C to 65°C then ramping to 95°C, followed by the final cooling to 50°C for 30 s. *PsACT* (Knopkiewicz and Wojtaszek, 2019) was used as the internal control. The expression level of each *PsRBOH* gene was calculated using the delta-delta Ct (2^−ΔΔCT^) method (Livak and Schmittgen, 2001). All analyses were conducted in three biological replicates and three technical replicates. All primer sequences used in this study were designed by Primer Premier 6.0 and are listed in **Supplementary Table S1**.

### Subcellular localization analysis

The coding sequence (CDS) of RBOH6 was amplified from the ZW6 and fused to the N terminus of the green fluorescent protein (GFP) in the pAN580 (GFP) vector. To determine the subcellular localization of RBOH6, the RBHO6-GFP vector was transiently co-expressed with endoplasmic reticulum (ER) marker mCherry-HDEL into *Arabidopsis* protoplasts (Ishikawa et al., 2018). And, the FM4-64 dye was used to marked plasma membrane. The fluorescent signals were observed using a confocal microscope (Leica SP8, Germany). Fluorescence signals were detected using the following excitation and emission wavelengths: GFP (488 nm/507 nm), FM4-64 (515 nm/640 nm) and mCherry (587 nm/610 nm).

## Results

### Chromosomal localization analysis of the *RBOH* gene family in pea

**Figure 1:**
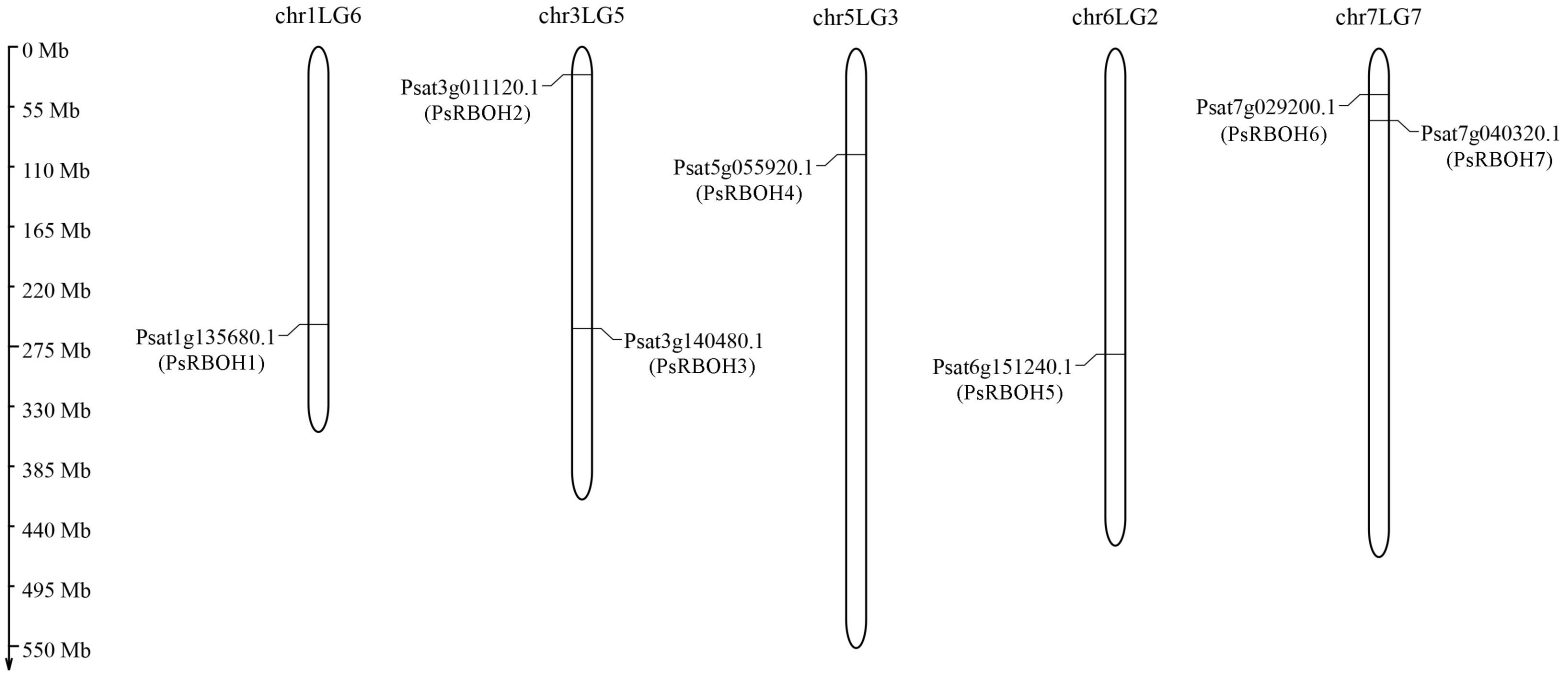
Genomic distribution of the *RBOH* genes in pea chromosomes.

Seven putative *PsRBOH* genes were retrieved from the pea genome after removing the redundant and repeat sequences. We named these *PsRBOH1*–*7* (**Figure 1** and **Supplementary Table S2**) based on their localization in the *P*. *sativum* genome. The distribution of these *RBOH* genes did not show a certain regularity. There was one *RBOH* gene on chromosomes chr1LG5 (*RBOH1*), chr5LG3 (*RBOH4*), and chr6LG2 (*RBOH5*) each, while two were on chromosomes chr3LG5 (*RBOH2* and *3*) and chr7LG2 (*RBOH6* and *7*) each.

### Phylogenetic analysis of the *PsRBOHs*

**Figure 2:**
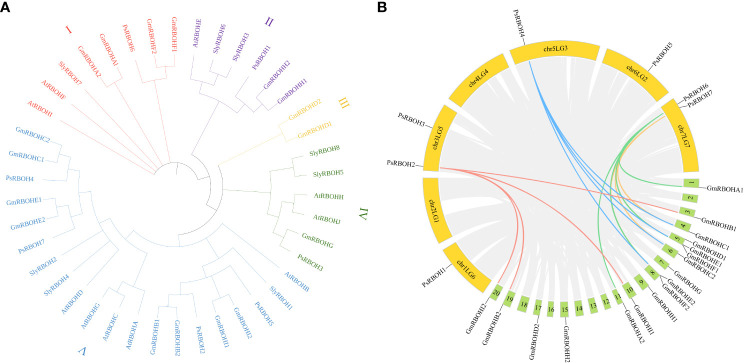
Phylogenetic and collinearity analyses of the *PsRBOHs*. **(A)** Phylogenetic analysis of *RBOHs* of pea, soybean, tomato, and *Arabidopsis*. **(B)** Collinearity analysis of *RBOHs* between pea and soybean. The colored lines represent *RBOHs* collinearity between species, and the gray lines are collinearity of all gene members between species. Yellow and green boxes represent pea and soybean chromosomes, respectively.

To investigate the evolutionary relationships of *RBOHs* among pea, soybean, tomato, and *Arabidopsis*, we constructed a neighbor-joining phylogenetic tree using the alignments of seven pea RBOH proteins, 17 soybean RBOH proteins, eight tomato RBOH proteins, and ten *Arabidopsis* RBOH proteins (**Figure 2A**). The results showed that 42 RBOHs from pea, soybean, tomato, and *Arabidopsis* were divided into five groups (I, II, III, IV, and V). Groups I, II, III, IV, and V contained 8, 6, 2, 6, and 20 RBOH proteins, respectively, and 1, 1, 0, 1 and 4 PsRBOHs were clustered in groups I, II, III, IV, and V, respectively.

### Collinearity analyses of the *PsRBOHs*

To explore the evolution of RBOH genes, we analyzed the synteny relationship of RBOHs between pea and soybean (**Figure 2B**). Four ortholog pairs were identified between pea and soybean. *PsRBOH2* paired with *GmRBOHB1*, *GmRBOHB2*, *GmRBOHI1*, and *GmRBOHI2*, while *PsRBOH4* paired with *GmRBOHC1*, *GmRBOHC2*, *GmRBOHE1*, and *GmRBOHE2*. *PsRBOH6* paired with *GmRBOHA1*, *GmRBOHA2*, *GmRBOHF1*, and *GmRBOHF2*, and *PsRBOH7* paired with *GmRBOHC1*, *GmRBOHC2*, *GmRBOHE1*, and *GmRBOHE2*.

### Gene structure, conserved motif and conserved domain analysis of the *PsRBOHs*

**Figure 3:**
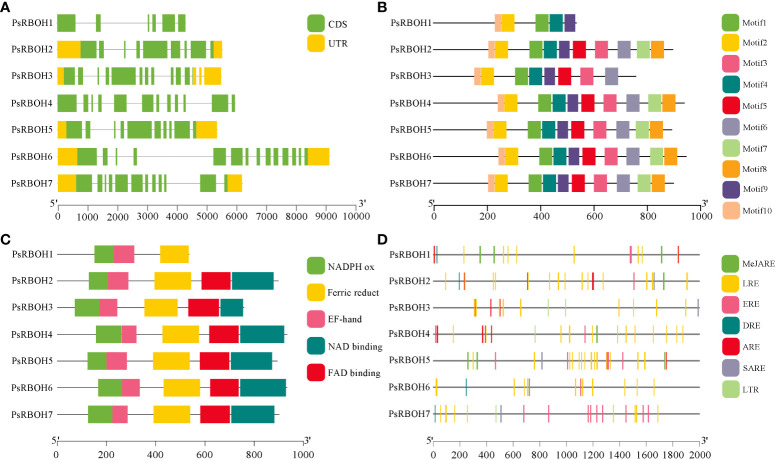
The gene structures, conserved motifs, conserved domain, and cis-elements analysis of putative promoters of PsRBOHs. **(A)** Gene structure of *PsRBOHs*. The green block represents the coding sequence (CDS), the yellow block shows the 5’ or 3’ untranslated regions (UTRs), and the black line indicates the intron. The scale bar indicates the length of the DNA sequences. **(B)** The conserved motifs in PsRBOHs were identified by MEME tools. Each motif is indicated by a rectangular box of a different color and the motifs are numbered from 1 to 10. The black line scale represents the length of amino acids. **(C)** Conserved domain compositions of PsRBOHs. Only major domains are presented in the figure based on our searches in the Pfam database. The black line scale represents the length of amino acids. **(D)** Cis-elements analysis of putative promoters of *PsRBOHs*. The black line scale represents the length of promoter amino acids. The cis-elements are as follows: MeJA-responsive elements (MeJARE), light-responsive elements (LREs), ethylene-responsive elements (EREs), drought-inducible elements (DRE), abscisic acid-responsive element (ARE), salicylic acid-responsive elements (SARE), and low temperature-responsive element (LTR).

A gene structure map of *PsRBOHs* was constructed based on the pea genome sequence (**Figure 3A**). The *PsRBOHs* had varying structures with different numbers of untranslated regions (UTRs) and exons. All *PsRBOHs* but *PsRBOH1* and *PsRBOH4* had no UTRs, and the number of exons ranged from six (*PsRBOH1*) to 14 (*PsRBOH6*). Ten conserved motifs were identified *via* the MEME online tool and were used to gain a deeper understanding of various motif compositions of the PsRBOHs. As shown in **Figure 3B** and **Supplementary Figure S1**, PsRBOH proteins contained 10 motifs, except PsRBOH1 and PsRBOH4, which had 4 and 9 motifs, respectively. Moreover, all PsRBOH proteins, except PsRBOH1, had four typical conserved domains, including the NADPH-Ox, Ferri-reduction, FAD-binding, and NAD-binding domains (**Figure 3C**). PsRBOH1 lacked the FAD-binding and NAD-binding domains, and all RBOH proteins contained the EF-hand domain.

### Cis-element analysis of the putative *PsRBOH* promoters

To further explore the function of *PsRBOH* genes in peas, we predicted the cis-elements of the promoters of these using the PlantCARE database (**Figure 3D**). The results showed that the light-responsive elements (LREs) were the most abundant (84) among the seven *PsRBOH* promoter sequences and widely distributed in all promoter sequences, followed by ethylene-responsive elements (EREs) (20), which was also distributed in all promoter sequences (**Figure 3D**). We also found that the promoter sequences of *PsRBOH* genes contained three hormone-responsive elements, [abscisic acid-responsive element (ARE, 17), MeJA-responsive element (MeJARE, 16) and salicylic acid-responsive elements (SARE, 5)] and two abiotic response elements [drought-inducible elements (DRE, 4) and low temperature-responsive element (LTR, 4)].

### Tissue-specific expression of *PsRBOHs* in pea

**Figure 4:**
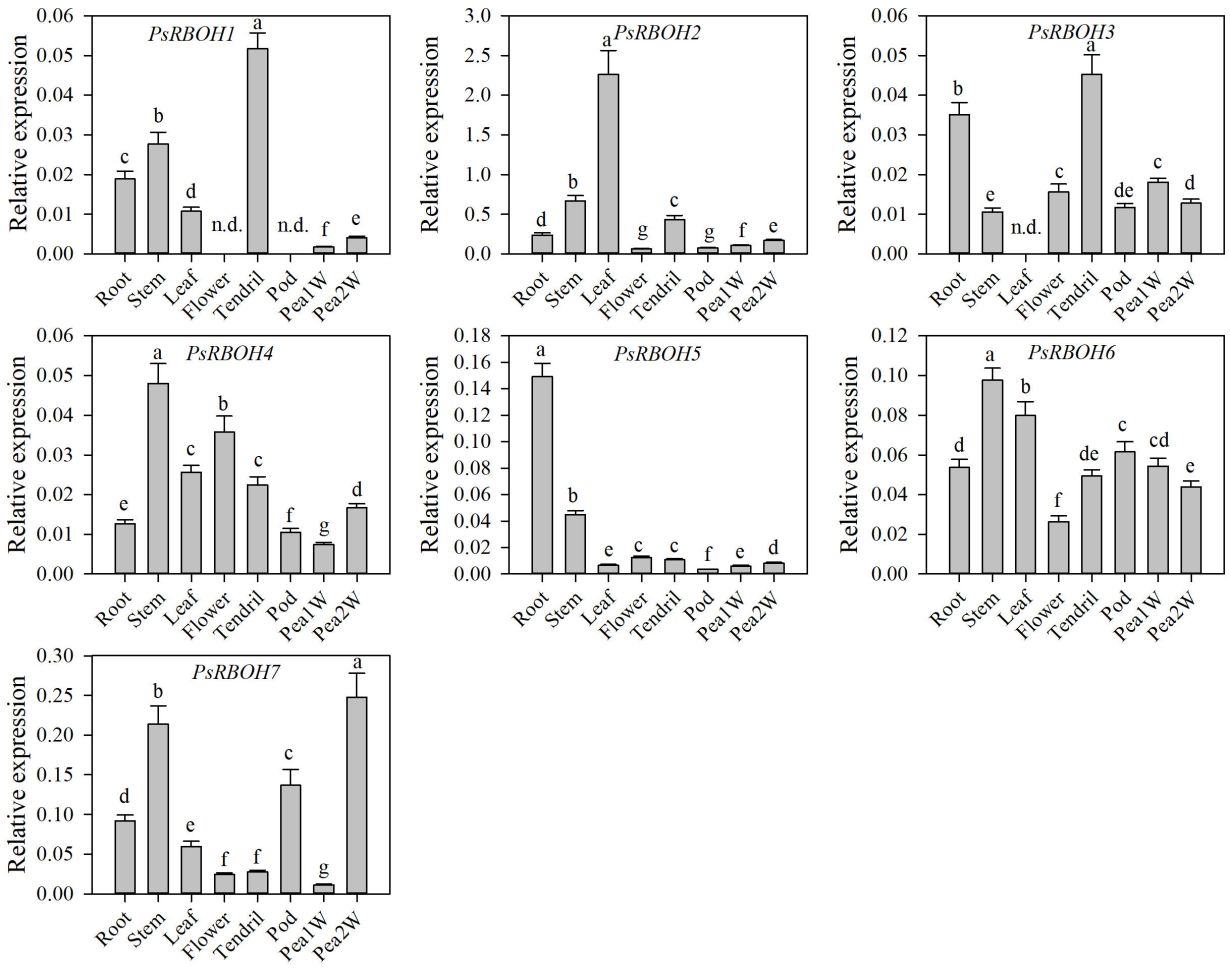
Expression patterns of *PsRBOH* genes in different tissues. Pea1W: One-week-old pea seeds; Pea2W: Two-week-old pea seeds. Error bars represent standard deviations for three biological replicates. Different letters indicating significant differences among tissues (*p*<0.05, Duncan’s test). n.d.: Not detected.

To determine the expression patterns of the *PsRBOH* genes in different tissues, we studied the tissue-specific transcriptional activity of seven genes in the root, stem, tendril, leaf, flower, pod, one-week-old pea seeds (Pea1W), and two-weeks-old pea seeds (Pea2W) of pea (**Figure 4**). The results showed that the expressions of the *PsRBOH* genes had significant differences in different tissues. *PsRBOH5* was highly expressed in the roots, while *PsRBOH4*, *PsRBOH6*, and *PsRBOH7* were highly expressed in the stem. *PsRBOH2* was highly expressed in all tissues. *PsRBOH6* was highly expressed in the leaves, but *PsRBOH3* was not detected in the leaves. Moreover, *PsRBOH1* and *PsRBOH3* were highly expressed in tendrils, and all seven *PsRBOH* genes had relatively lower expression levels in the pod and Pea1W. *PsRBOH5* and *PsRBOH7* were highly expressed in the roots and Pea2W, respectively, but *PsRBOH1* was not expressed in flowers and pods.

### Expression of *PsRBOHs* under different abiotic stresses

To further elucidate the expression patterns of pea *RBOH* genes under different abiotic stresses, we evaluated the expression profiles of *PsRBOHs* in the leaves and roots under heat stress, cold stress, salt stress, Cd stress, PEG-induced drought stress (PEG), and low-boron (LB) stress at various time points after the treatment.

**Figure 5:**
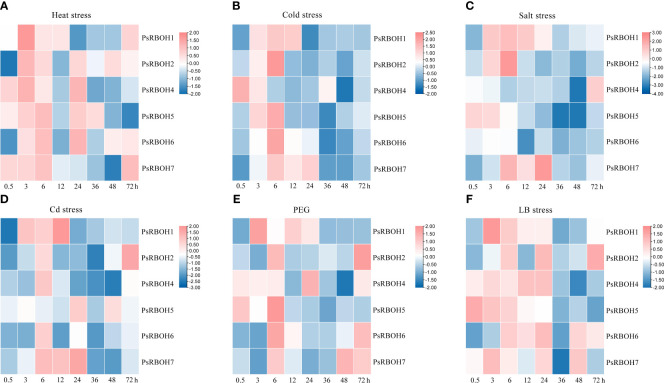
Leaf expression of *PsRBOHs* under heat stress **(A)**, cold stress **(B)**, salt stress **(C)**, cadmium (Cd) stress **(D)**, polyethylene glycol (PEG)-induced drought stress (PEG) **(E)**, and low boron (LB) stress **(F)**. The log-transformed relative expression levels were used to generate the heatmaps. The color scale is shown on the right.

The abiotic stresses induced or inhibited the expression of genes in a highly specific manner, with the most significant changes occurring at the early stages of stress treatment (**Figure 5**). The expression levels of all genes increased in the leaves in all six treatments (**Figures 5A–F**), except for *PsRBOH4* under salt stress (**Figure 5C**) and *PsRBOH5* under Cd stress (**Figure 5D**). All *PsRBOH* genes were up-regulated at 6 h under all treatments (**Figures 5A–F**) except for *PsRBOH4* under cold stress (**Figure 5B**), *PsRBOH5* under Cd stress (**Figure 5D**) and *PsRBOH4-6* under salt stress (**Figure 5C**). *PsRBOH1* was up-regulated at 3 h under all six treatments but down-regulated at 24 h under heat (**Figure 5A**), cold (**Figure 5B**) and Cd stresses (**Figure 5D**). *PsRBOH3* was not expressed in the leaves under all treatments (**Figures 5A–F**). Furthermore, *PsRBOH4* was down-regulated from 0.5 to 48 h but up-regulated at 72 h under salt stress (**Figure 5C**). *PsRBOH5* was induced at 0.5 h under heat stress (**Figure 5A**) but down-regulated at 12 h under heat (**Figure 5B**), salt (**Figure 5C**), Cd (**Figure 5D**), and PEG (**Figure 5E**) stresses. In addition, *PsRBOH6* was induced at 6 h under LB stress (**Figure 5F**) and down-regulated at all time points except 3 h and 6 h under salt stress (**Figure 5C**).

**Figure 6:**
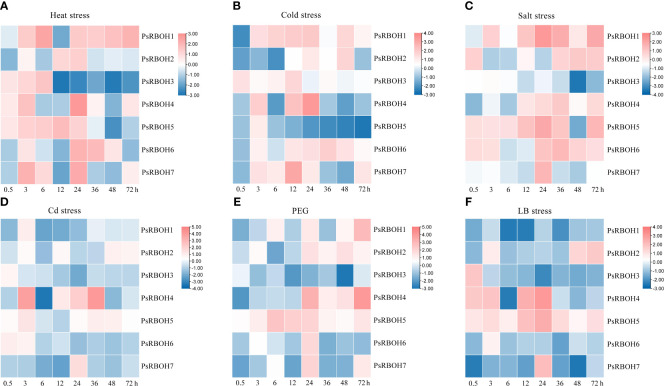
Root expression of *PsRBOHs* under heat stress **(A)**, cold stress **(B)**, salt stress **(C)**, cadmium (Cd) stress **(D)**, polyethylene glycol (PEG)-induced drought stress (PEG) **(E)**, and low boron (LB) stress **(F)**. The log-transformed relative expression levels were used to generate the heatmaps. The color scale is shown on the right.

The expression patterns of *PsRBOHs* had similarities and differences in the roots and leaves. In the roots, *PsRBOH5* was induced at 0.5 h under heat (**Figure 6A**), salt (**Figure 6C**), PEG (**Figure 6E**), and LB (**Figure 6F**) stresses and maintained the high expression until 72 h under salt (**Figure 6C**), PEG (**Figure 6E**) and LB (**Figure 6F**) stresses. *PsRBOH1* was up-regulated at 3 h and maintained a high expression level until 72 h under cold (**Figure 6B**) and salt stresses (**Figure 6C**) except at 12 h under cold stress. However, *PsRBOH1* was down-regulated at all time points under LB treatment (**Figure 6F**). *PsRBOH6* was induced at 0.5 h under salt (**Figure 6C**) and Cd (**Figure 6D**) treatments but down-regulated from 6 to 72 h under Cd (**Figure 6D**) and LB (**Figure 6F**) treatments. Additionally, *PsRBOH6* had a similar expression pattern with *PsRBOH1* under LB stress, except at the 3 h time-point (**Figure 6F**).

### Subcellular localization analysis

**Figure 7:**
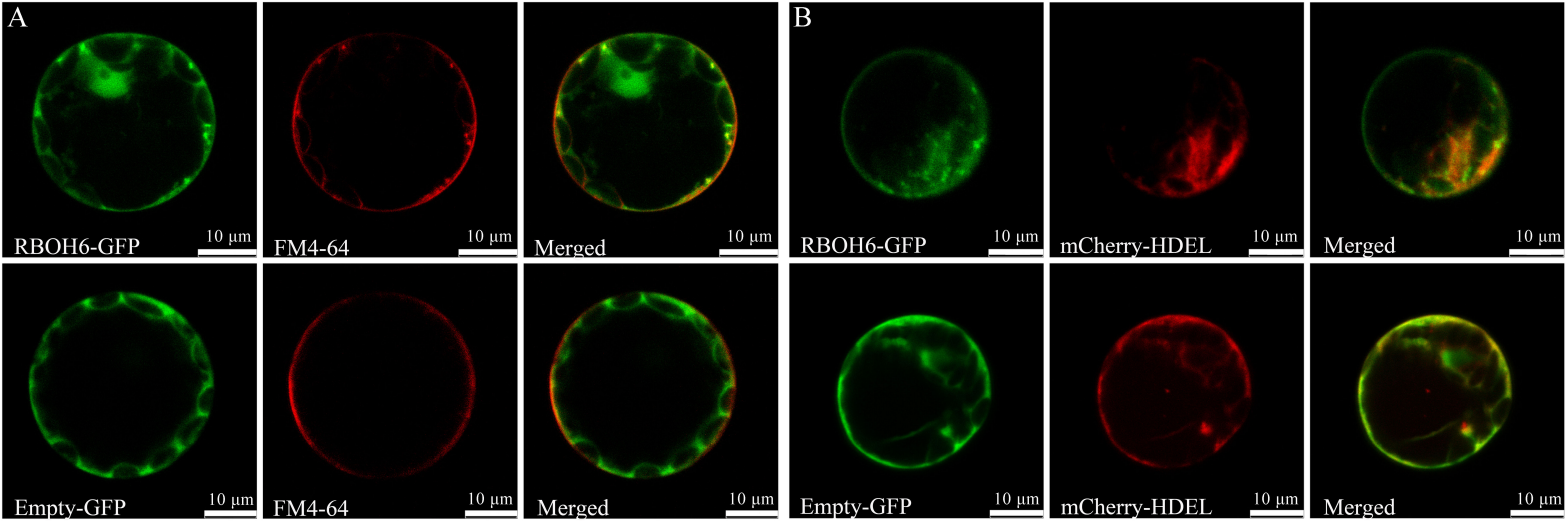
Subcellular localization of PsRBOH6 protein. **(A)** The plasma membrane was stained by FM4-64. **(B)** Transient co-expression of PsRBOH6-GFP with endoplasmic reticulum marker mCherry-HDEL. Empty vector was as control. Scale bar = 10 μm. Single optical sections obtained by CLSM.

The subcellular localization of pea RBOH proteins was predicted using WoLFPSORT (**Table 1**). The results indicated that four PsRBOH proteins were highly likely to be located in the plasma membrane. PsRBOH4 and PsRBOH5 were presumably located in the nucleus, and PsRBOH4 in the cytosol. Evolutionary analysis showed that PsRBOH6 was homologous to AtRBOHF and GmRBOHF1/2. It has been shown that multiple calcineurin B-like (CBL) interacting protein kinases (CIPKs) target *Arabidopsis* RBOHF by directly binding Ca^2+^ to its EF-hands to fine-tune superoxide production in response to different stimuli (Drerup et al., 2013; Han et al., 2019). Since AtRBOHF localizes in the plasma membrane (Drerup et al., 2013), PsRBOH6 was selected for a transient expression assay using *Arabidopsis* mesophyll protoplasts (**Figure 7**). The PsRBOH6-GFP plasmid was transiently co-expressed with the plasma membrane and endoplasmic reticulum markers, FM4-64 (**Figure 7A**) and mCherry-HDEL (**Figure 7B**), in *Arabidopsis* leaf protoplasts, respectively. As shown in **Figure 7**, GFP-PsRBOH6 fluorescent signals were extensively co-localized with FM4-64 and mCherry-HDEL, suggesting that PsRBOH6 localizes in the plasma membrane and endoplasmic reticulum.

**Table 1:** The detailed information of PsRBOH members.

Gene	Annotated	Genomic position	CDS	AA	Subcellular localization
PsRBOH1	Psat1g135680.1	chr1LG6:268211884- 268216192	1608	535	plas: 5, E.R.: 5, mito: 2, chlo: 1
PsRBOH2	Psat3g011120.1	chr3LG5:26369259-26374797	2694	897	cyto: 5, nucl: 3, plas: 3, chlo: 1, vacu: 1
PsRBOH3	Psat3g140480.1	chr3LG5:273226086-273231589	2277	758	plas: 8, E.R.: 2, chlo: 1, nucl: 1, mito: 1
PsRBOH4	Psat5g055920.1	chr5LG3:101924110-101930071	2823	940	nucl: 5, plas: 3, cyto: 2, chlo: 1, vacu: 1, E.R.: 1
PsRBOH5	Psat6g151240.1	chr6LG2:294961062-294966418	2682	893	nucl: 7, cyto: 3, plas: 2, chlo: 1
PsRBOH6	Psat7g029200.1	chr7LG7:45297780-45306906	2844	947	plas: 11, nucl: 3
PsRBOH7	Psat7g040320.1	chr7LG7:69319366-69325556	2703	900	plas: 9, cyto: 4

Chr, chromosome; CDS, length of coding sequence; AA, number of amino acids; The subcellular location of pea RBOH proteins was predicted using WoLF PSORT (http://www.genscript.com/psort/wolf_psort.html). Nucl, nucleus; Mito, mitochondria; Chlo, chloroplast; Cyto, cytosol; E.R, endoplasmic reticulum; Plas, plasma membrane.

## Discussion

*RBOH* genes are involved in plant signal transmission, morphogenesis and development, and responses to biotic and abiotic stresses. However, the *RBOH* gene family has been identified in various plants but not in pea. These studies will be useful for understanding the role of *RBOH* genes in plant responses to abiotic stresses.

The present study analyzed the *RBOH* gene family in pea and identified seven *RBOH* genes were identified in the pea genome. The identified *RBOH* genes were fewer than those of *Arabidopsis* (10) (Sagi and Fluhr, 2006), rice (9) (Groom et al., 1996), *Pyropia yezoensis* (11) (Gui et al., 2022), *Solanum melongena* (8) (Du et al., 2023), and soybean (17) (Liu et al., 2019), but same those of strawberry (7) (Zhang et al., 2018) and alfalfa (7) (Marino et al., 2011). The chromosomal distribution of *RBOH* genes did not show a certain regularity, the same as in other plants (Selvi et al., 2020; Gui et al., 2022; Du et al., 2023). Gene structure analysis revealed that the number of *PsRBOH* exons varied between 6-16 and harbored in 9-12, similar to that of *Arabidopsis* and rice, which were harbored in 10-14 (Zhang et al., 2018). *PsRBOH1* had the lowest number of exons (6), while *PsRBOH6* had the most exons (16), indicating that *PsRBOHs* experienced both conservation and diversification during their evolution.

Determining the phylogenetic relationships among species is fundamental for many biological studies (Kapli et al., 2020). Phylogenetic analysis indicated that members of the *RBOH* gene families in pea, soybean, tomato, and *Arabidopsi*s could be divided into five groups, indicating that RBOHs are conserved in various species (Chang et al., 2020; Chen and Yang, 2020; Gui et al., 2022; Du et al., 2023).

In soybeans, the ancestral *Papilionoideae* whole-genome duplication event and *Glycine*-specific duplication event resulted in nearly 75% of the genes present in multiple copies (Schmutz et al., 2010). While there has no a recent whole-genome duplication but reflects the ancestral *Papilionoideae* whole-genome duplication event in pea (Kreplak et al., 2019). This also explains why the amount of *RBOHs* in pea is less than that in soybean and the collinear gene pairs of four *PsRBOHs* (*PsRBOH2*, *PsRBOH4*, *PsRBOH6*, and *PsRBOH7*) in soybean.

In *Arabidopsi*s, all ten RBOHs had four typical conserved domains: NADPH-Ox, Ferri-reduction, FAD-binding, and NAD-binding domains (Sagi and Fluhr, 2006). However, in wheat, 4 out of 36 NADPH oxidases had the NADPH_Ox domain but lacked one or two other conserved domains (Hu et al., 2018). In eggplant, 5 out of 8 *SmRBOHs* lacked the FAD-binding domain, which was substituted by the NOX_Duox_like_FAD_NADP domain (Du et al., 2023). In this study, all PsRBOHs, except PsRBOH1, had the four typical conserved domains. PsRBOH1 lacked the FAD-binding and NAD-binding domains which are crucial for electron transfer and ROS production (Wang et al., 2022). Therefore, the possible function of RBOH1 in the production of superoxide needs further verification.

The tissue-specific expression of genes is crucial for plant growth and development and provides important insights into understanding gene function. The tissue-specific expression patterns of the *RBOHs* have been reported in many plants; however, the expression patterns are distinct in different plants. Two out of ten, four out of seven, and all seven *RBOH* genes were expressed throughout *Arabidopsis* (Sagi and Fluhr, 2006), strawberry (Zhang et al., 2018) and grape (Cheng et al., 2013), respectively. However, only four out of ten *AtRBOH* genes were specifically expressed in the roots and elongation zone (Sagi and Fluhr, 2006). In this study, five *RBOHs* (*PsRBOH2* and *PsRBOH4-7*) were expressed in all tissues, while *PsRBOH1* was not detected in the flowers and pods, and *PsRBOH3* was not expressed in the leaves. In addition, the expression of *PsRBOH2* was higher in all tissues, especially in the leaves. In soybeans, *GmRBOHB1*, which was orthologous with *PsRBOH2* and clustered in one cluster, was highly expressed in the leaves (Liu et al., 2019). These results indicated the tissue specificity of the *PsRBOH* genes, suggesting that these genes may have different functions during plant development.

Cis-acting elements regulate the expression of target genes by binding the trans-acting factors (Yamaguchi-Shinozaki and Shinozaki, 2005). It has been reported that *RBOH* family members in various plants are induced by different abiotic stress stimuli, such as drought (Duan et al., 2009), salt (Xie et al., 2011), heat (Li et al., 2014; Xia et al., 2014), wounding (Sagi et al., 2004), and cold stress (Zhang et al., 2018). Moreover, *RBOHs* respond to environmental stimuli through hormonal signaling networks involving abscisic, salicylic, jasmonic acid, and ethylene (Overmyer et al., 2003). In the present study, many hormone-responsive elements (such as ARE, ERE, MeJARE, and SARE) and abiotic response elements (such as DRE and LTR) were found in the promoter of *PsRBOHs*, indicating their potential roles in pea response to phytohormones and stresses.

The involvement of the *RBOH* gene family members in abiotic stress responses has been reported in many plants. In grapes, *VvRBOHs* were significantly increased under salt, drought, powdery mildew, salicylic acid, and abscisic acid treatments (Cheng et al., 2013). It was reported that the expression level of *AtRBOHD* was significantly increased in *Arabidopsis* at an early stage in response to hypoxia (Sagi and Fluhr, 2006). Soybean *RBOH* genes were significantly induced by salt, PEG, cold, and Cu stresses, and *GmRBOHD2* was significantly induced by salt, polyethylene glycol, low temperature, and Cu toxicity in the roots (Liu et al., 2019). In the present study, all *PsRBOH* genes responded to all six stresses. Moreover, *PsRBOH3*, which had a close evolutionary relationship with *GmRBOHD2*, was expressed in the roots and not in leaves, and its expression occurred earlier (0.5 - 6 h) after heat, cold, and LB stress treatments. The expression of *PsRBOH1* was up-regulated in the leaves 3 h after the abiotic stress treatments and lasted for up to 24 h, after which it was down-regulated under heat, cold, salt and Cd treatments. However, *PsRBOH1* was up-regulated in the roots by heat, cold and NaCl treatments but down-regulated by LB treatment. A similar phenomenon was also observed in cotton (Wang et al., 2020). These results indicate that the *PsRBOH* gene family could be involved in the abiotic stress response, but the regulatory mechanisms are still unclear.

Superoxide can be synthesized in different cellular compartments, such as chloroplasts (Foyer and Hanke, 2022), mitochondria (Postiglione and Muday, 2022), peroxisome (Sandalio et al., 2021), and the plasma membrane oxidoreductase system (Lherminier et al., 2009; Jiménez-Quesada et al., 2022; Miller and Mittler, 2023). In leaves from pea plants grown with 50 µm CdCl_2_, the accumulation of H_2_O_2_ was observed mainly in the plasma membrane of transfer, mesophyll and epidermal cells, as well as in the tonoplast of bundle sheath cells (Romero-Puertas et al., 2004). RBOH is present in the plasma membrane systems of almost all animals and plants (Chen and Yang, 2020). Subcellular location is a key characteristic that determines the function of many proteins, indicating that proteins in different subcellular locations have different functions (Koroleva et al., 2005). All the RBOHs in *Arabidopsis* (Sagi and Fluhr, 2006), rice (Groom et al., 1996) and wheat (Hu et al., 2018) were predicted to localize to the plasma membrane. Some RBOHs were shown to localize to the chloroplast thylakoid membrane of grapes (Cheng et al., 2013) and strawberry (Zhang et al., 2018). Moreover, in *Gossypium* barbadense, 71 out of 87 RBOHs were located in the cytoplasm (Chang et al., 2020). Tobacco RBOHD was localized in the plasma membrane and Golgi cisternae (Noirot et al., 2014). In *P. vulgaris*, PvRBOHA was localized in the plasma membrane of root hair (Arthikala et al., 2017), whereas PvRBOHB was localized in the central apical dome (Montiel et al., 2012). These different subcellular distributions of RBOHs in root hairs resulted in two models that explain root hair development (Arthikala et al., 2017). In pea, four out of the seven RBOHs had a high probability of being localized in the plasma membrane, indicating similar functions. Two of the PsRBOHs were presumably located in the nucleus, while PsRBOH6 was localized in the cell membrane and endoplasmic reticulum, suggesting that they may regulate ROS production at different subcellular locations (Lee et al., 2023).

## Conclusions

This study comprehensively analyzed the *RBOH* gene family in the pea genome. Seven *PsRBOH* genes were identified and were divided into five groups, which were distributed on five chromosomes. In addition, we analyzed collinearity, gene structure, conserved domains, conserved motifs, cis-elements, and subcellular distribution of the *PsRBOH* genes. The *PsRBOHs* exhibited tissue specificity and functional diversity during plant growth and response to different abiotic stresses. Overall, these results provide valuable information which could be used for further functional analysis of pea *RBOH* genes in response to climate-driven environmental constraints.

## Data availability statement

The original contributions presented in the study are included in the article/**Supplementary Material**. Further inquiries can be directed to the corresponding authors.

## Author contributions

ML: Formal Analysis, Visualization, Writing – original draft, Writing – review & editing, Conceptualization, Funding acquisition, Supervision. YZ: Formal Analysis, Visualization, Writing – original draft, Validation. TP: Formal Analysis, Writing – review & editing. YL: Investigation, Writing – review & editing. YH: Investigation, Writing – review & editing. WC: Investigation, Writing – review & editing. YY: Investigation, Writing – review & editing. GZ: Writing – review & editing, Formal Analysis, Visualization, Writing – original draft. SS: Writing – review & editing. MY: Writing – review & editing.

## Supplementary material

Supplementary Figure 1 Amino acid sequence of predicted motif.
