# Supplementary figures and images for: Genome-wide analysis of respiratory burst oxidase homolog gene family in pea (Pisum sativum L.)
Source: Front Plant Sci. 2023 Dec 12;14:1321952. doi: 10.3389/fpls.2023.1321952 (PMC10754532; doi:10.3389/fpls.2023.1321952)

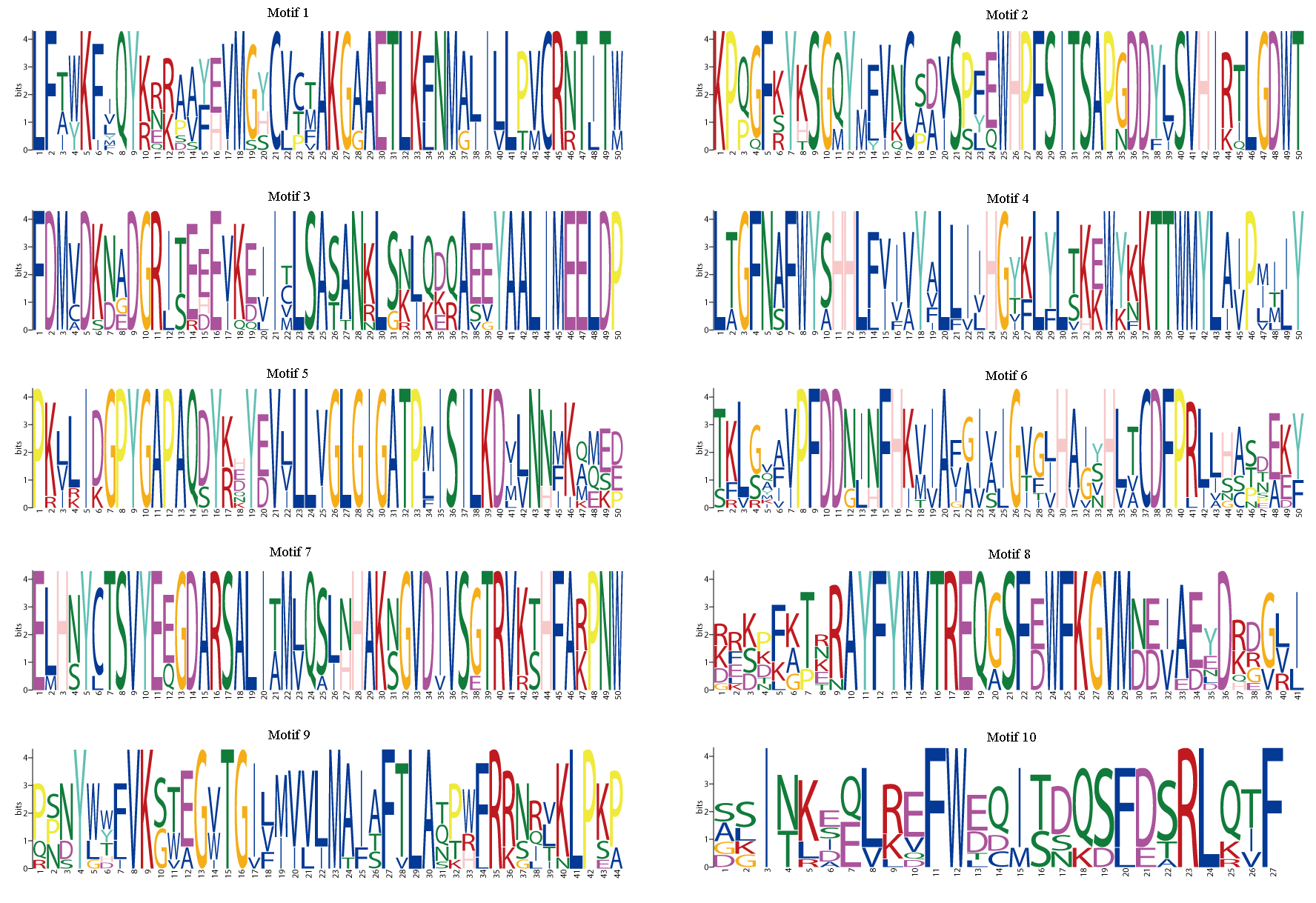

Supplement: Supplementary Figure 1 — Amino acid sequence of predicted motif. [file DataSheet_1.zip › Data Sheet 1/Supplementary Figure S1.jpg]
